# Identification of the Photoreceptor Transcriptional Co-Repressor *SAMD11* as Novel Cause of Autosomal Recessive Retinitis Pigmentosa

**DOI:** 10.1038/srep35370

**Published:** 2016-10-13

**Authors:** M. Corton, A. Avila-Fernández, L. Campello, M. Sánchez, B. Benavides, M. I. López-Molina, L. Fernández-Sánchez, R. Sánchez-Alcudia, L. R. J. da Silva, N. Reyes, E. Martín-Garrido, O. Zurita, P. Fernández-San José, R. Pérez-Carro, F. García-García, J. Dopazo, B. García-Sandoval, N. Cuenca, C. Ayuso

**Affiliations:** 1 Department of Genetics & Genomics, Health Research Institute–Jiménez Díaz Foundation University Hospital (IIS-FJD), Madrid, Spain; 2 Centre for Biomedical Network Research on Rare Diseases (CIBERER), ISCIII, Madrid, Spain; 3 Department of Physiology, Genetics and Microbiology, University of Alicante, Alicante, Spain; 4 Department of Ophthalmology, Health Research Institute– Jiménez Díaz Foundation University Hospital (IIS-FJD), Madrid, Spain; 5 Universidade de Mogi das Cruzes, São Paulo, Brazil; 6 Computational Genomics Department, Centro de Investigación Príncipe Felipe (CIPF), Valencia, Spain; 7 Bioinformatics in Rare Diseases (BIER), Centre for Biomedical Network Research on Rare Diseases (CIBERER), Valencia, Spain; 8 Functional Genomics Node (INB), Valencia, Spain

## Abstract

Retinitis pigmentosa (RP), the most frequent form of inherited retinal dystrophy is characterized by progressive photoreceptor degeneration. Many genes have been implicated in RP development, but several others remain to be identified. Using a combination of homozygosity mapping, whole-exome and targeted next-generation sequencing, we found a novel homozygous nonsense mutation in *SAMD11* in five individuals diagnosed with adult-onset RP from two unrelated consanguineous Spanish families. SAMD11 is ortholog to the mouse major retinal SAM domain (mr-s) protein that is implicated in CRX-mediated transcriptional regulation in the retina. Accordingly, protein-protein network analysis revealed a significant interaction of SAMD11 with CRX. Immunoblotting analysis confirmed strong expression of SAMD11 in human retina. Immunolocalization studies revealed SAMD11 was detected in the three nuclear layers of the human retina and interestingly differential expression between cone and rod photoreceptors was observed. Our study strongly implicates *SAMD11* as novel cause of RP playing an important role in the pathogenesis of human degeneration of photoreceptors.

Retinitis Pigmentosa (RP, [MIM #268000]) is the most frequent cause of inherited retinal dystrophy (IRD), with an estimated global incidence of 1:4000 individuals^1^. This condition is characterized by progressive loss of photoreceptor function and viability, ultimately leading to blindness. Subjects diagnosed with RP initially complain of night blindness and progressive peripheral constriction of their visual field due to primary rod photoreceptor dysfunction. Central vision loss is also frequently presented as a secondary outcome in advanced disease course due to cone photoreceptor involvement. Large phenotypic variations have been reported between individuals, with a variable onset of the disease from childhood to adulthood^2^.

RP is inherited in most cases as a Mendelian trait: autosomal recessive in 30% of patients, autosomal dominant in 20% and X-linked in 10%. Approximately 40% of RP patients represent isolated cases^3^,^4^. A remarkable characteristic of RP is their enormous allelic and genetic heterogeneity. To date, more than 3,000 mutations in at least 60 genes have been reported to cause non-syndromic autosomal recessive RP (arRP)^5^, most of which are mutated only in a small fraction of patients. Combining Sanger sequencing and targeted-capture next-generation sequencing (NGS), it is possible to identify underlying causative mutations in 40–70% of arRP cases^6^,^7^,^8^ which implies that additional genes have yet to be identified. To shed light on novel autosomal recessive RP genes, we focused on whole-exome sequencing (WES) in Spanish families with evidence of parental inbreeding who did not carry any mutation in known IRD genes after whole genome homozygosity mapping. Using this strategy, we identified recently two novel genes, *ABHD12* and *ZNF408*, associated with non-syndromic arRP in our cohort of patients^9^,^10^.

Herein, we reported a homozygous nonsense mutation in *SAMD11* in five patients diagnosed with RP, providing first link between this gene and a retinal disorder. Human *SAMD11* is the human ortholog of the mouse major-retinal SAM domain (mr-s) gene, which is predominantly expressed in developing retinal photoreceptors^11^. Here, we determined for the first time the neural localization pattern of SAMD11 in the adult human retina. Thus, we observed a strong expression of SAMD11 in photoreceptor cells. Our findings allowed the identification of a new candidate gene underlying RP and provide insight into the *SAMD11* dysfunction in human retinal degeneration.

## Results

### Whole-genome homozygosity mapping

**Figure 1:**
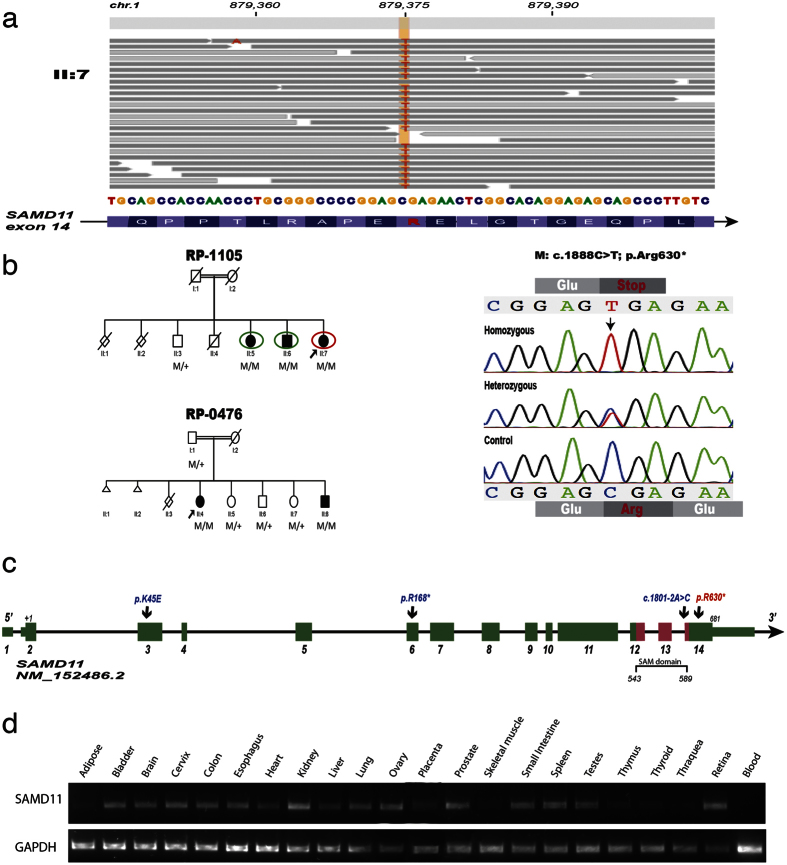
Identification of the homozygous nonsense mutation p.Arg630* associated to autosomal recessive Retinitis Pigmentosa by combining homozygosity mapping and whole-exome sequencing. (**a**) Mapped reads from the whole-exome sequencing (WES) analysis in patient II:7 from Family RP-1105 revealed a homozygous change C>T at position 879375 on chromosome 1, leading to a stop gain p.Arg630* in the *SAMD11* gene. Wild-type sequence and coverage per base are shown. (**b**) Pedigree of the two families carrying the p.Arg630* mutation in *SAMD11*, along with the correctly segregation of the mutant allele with a recessive inheritance. Individuals surrounded by a circle were analysed by homozygosity mapping using genome-wide SNP arrays. The red circle indicates the individual in which WES has been performed. The *SAMD11* genotype of each available family member is represented below the individual symbol being “+” normal allele and “M”, mutated alleles. Electropherograms of homozygous affected, heterozygous carrier and a healthy control subject for the c.1888C>T variant were also shown. (**c**) Intron-exon structure of *SAMD11* and position of novel likely pathogenic variants identified in this study. Exons are indicated by coloured rectangles that are wider for the coding regions. Exons in red encode the evolutionary conserved SAM domain of the SAMD11 protein. Nucleotide numbering reflects cDNA in the reference sequence NM_152486.2. (**d**) Expression of *SAMD11* by RT-PCR analysis in total RNA from 22 different human tissues. Amplification of *GAPDH* mRNA was used as positive control.

Three affected siblings (II:5, II:6 and II:7) of a consanguineous Spanish family (Family RP-1105, Fig. 1b) were diagnosed with autosomal recessive adult-onset RP. To identify the genetic cause underlying the arRP within the family, first we performed whole genome homozygosity mapping using high resolution SNP-array in each of the three affected siblings (II:5, II:6 and II:7) using Illumina HumanCytoSNP-12 SNP microarrays. Three regions of homozygosity >1 Mb were shared by all affected individuals, containing a total of 302 genes (Supplementary Table S1): a 20.4 Mb interval on chromosome 3 and two intervals of 11.8 and 1.3 Mb on chromosome 1. *CLRN1* was the only IRD-associated gene^12^,^13^ to be present within the candidate identity-by-descent (IBD) regions; however causal mutations were discarded by Sanger sequencing.

### Exome-sequencing detects a novel homozygous nonsense mutation in SAMD11

To analyse the above IBD candidate regions in this family, we performed whole-exome sequencing in the index case. A total of 69,657,399 reads were uniquely mapped to the exonic regions with a median of coverage of 86.25X. A total of 7,240 single nucleotide variations (SNVs) and 285 small insertions and deletions (INDELs) were identified by GATK program (Supplementary Table S2). Among them, 296 novel or rare variants were selected by excluding referenced polymorphisms with a minor allele frequency (MAF) >0.5% at dbSNP, 1000 genomes^14^ and Exome Variant Server (EVS)^15^ databases. No pathogenic variants were found in the more than 200 genes previously implicated in IRD. Under the assumption of recessive inheritance and consanguineous ancestry, homozygous variants within the previously candidate IBD regions were prioritized, remaining only two novel variants, both located at the third shared region on the short arm of chromosome 1 (1p36.33): i) a nonsense variant (NM_152486.2:c.1888C>T;p.Arg630*) in *SAMD11* (Sterile alpha motif domain-containing 11) and ii) a missense change (NM_032129.2: c.995G>A; p.Gly332Glu) in *PLEKHN1 (Pleckstrin homology domain containing, family N member 1)* (Supplementary Tables S2 and S3). Both variants were validated by Sanger sequencing, segregating homozygously with the disease in the family (Fig. 1b) and were excluded from 196 ethnically matched-control individuals. In addition, they have not been described either in the Exome Agregation Consortium (ExAC) database or in an in-house 400 Spanish exomes database (CIBERER Spanish Variant Server).

The nonsense variant p.Arg630* at *SAMD11* appeared to be the top candidate based on potential deleterious effect predicted by several *in silico* prediction tools, and biological and clinical relevance according to gene function, expression and *in silico* protein interaction network analysis with other known IRD genes^16^,^17^ (Supplementary TableS3). This gene is predominantly expressed in photoreceptor cells^11^. This mutation introduces a premature termination codon (PTC) at the last exon of *SAMD11,* truncating the last C-terminal 50 residues. *In silico* protein-protein interaction analysis revealed a network with a clustering coefficient significantly higher than expected by chance (*p-value* = 0.0022) that interestingly depicts relationships of *SAMD11* to several retinal dystrophy-associated genes such as the photoreceptor-specific transcription factor Cone-Rod homeobox (*CRX*) (Supplementary Figure S1). By contrast, the second novel variant found in this family was a novel missense p.Gly332Glu in *PLEKHN1,* a gene with unknown function. This variant was predicted to be likely deleterious by several *in silico* tools (Supplementary Table S3), however a clear correlation of this gene with IRD could not be inferred.

### Mutational screening in additional IRD cohorts

To further evaluate if these variants might be also present in other RP patients, both variants were also screened in 380 unrelated Spanish index cases suffering from autosomal recessive or sporadic RP (SRP). Remarkably, the p.Arg630* variant was also found homozygously in a second consanguineous pedigree (RP-0476, Fig. 1B) with two siblings suffering with adult-onset RP, and correctly segregated according to a recessive inheritance pattern in the family. The p.Arg630* mutation in *SAMD11* seems not to be ancestrally inherited in both unrelated families as demonstrated by haplotype analysis (Supplementary Figure S2). In view of these evidences, the identification of a nonsense mutation in 5 affected subjects from two unrelated families reinforces a very likely pathogenic role of *SAMD11* in the RP development.

To determine whether mutations in *SAMD11* could be a common cause of IRD, this gene was exhaustively screened in additional 400 Spanish IRD patients using Sanger sequencing or customized targeted NGS approach^18^,^19^. In addition, genome-wide homozygous regions from 300 unrelated individuals with several autosomal recessive IRDs were also assessed through the European Retinal Disease Consortium (ERDC)^20^. Two families presented large IBD regions encompassing the *SAMD11* locus and were also screened for mutations by Sanger sequencing.

During those screenings, we have additionally identified three novel likely pathogenic variants in *SAMD11* (Supplementary Table S4), all carried in heterozygosis (Fig. 1b), including one nonsense mutation (c.502C>T; p.Arg168*), one splice site variant at intron 13 (c.1801–2A>C) and one missense (c.133A>G; p.Lys45Glu) variant. These novel variants were not present in any SNV database neither in 196 Spanish control individuals nor in our 400 in-house whole-exome dataset. The variant p.Lys45Glu affected a highly evolutionary conserved amino-acid and was predicted as a very likely pathogenic variant by several *in silico* predictor tools (Supplementary Table S5). Large rearrangements, small exon deletions or large copy number variations (CNVs) affecting the *SAMD11* gene, were discarded in patients carrying a heterozygous likely pathogenic variant using a custom-designed high-resolution comparative genomic hybridization (CGH) array (Supplementary Figure S3).

### Ophthalmic examination

**Figure 2:**
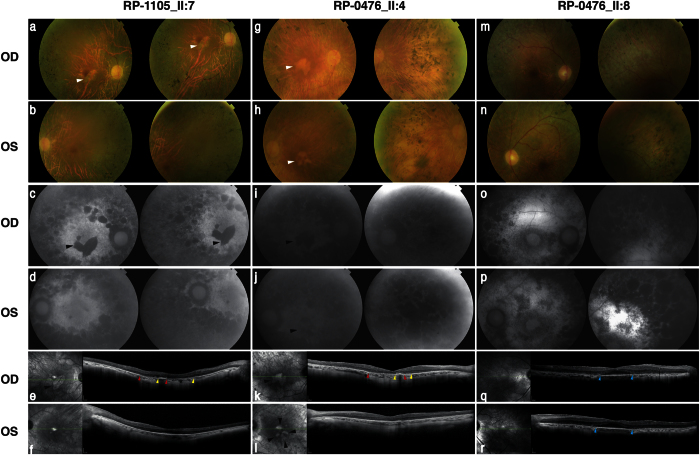
Retinal imaging of patients carrying the p.Arg630* mutation in *SAMD11*. Data from the index case (II:7) from family RP-1105 and the two affected siblings (II:4 and II:8) from family RP-0476 were included. **(a–d)** Fundus imaging of the right and left eye of individual II:7 at 66 years of age. (**a,b**) Fundus color photographs, showing pigmentary changes, retinal pigment epithelium (RPE) atrophy in the mid-periphery of both eyes and also atrophic changes in the macula (white arrowheads). (**c,d**) Fundus autofluorescence (FAF) showed a marked hypofluorescence area below the fovea manifesting the existence of a significant atrophic plaque in right eye (OD, black arrowhead), while it is not observed in the left eye (OS). **(e,f**) Macular optical coherence tomography (OCT), confirming the RPE loss in fovea and destructuring of outer layers in OD, and conservation of RPE and outer segment in fovea in OS. On the right image, black arrowheads indicate the localization of the atrophic plaques in parafoveal region. Red and yellow arrowheads indicate start and end position, respectively for atrophic RPE loss in OD. **(g–j)** Fundus imaging of the right and left eye of the index case II:4 from family RP-0476 at 74 years-old. (g-h) Fundus color photographs showing RPE alteration in paravascular areas and mid-periphery, round and bone-spicule pigmentation in mid-periphery, attenuated retinal vessels and central atrophic plaques (white arrowheads). (**i,j**) FAF, confirming also the presence of hypofluorescence regions at fovea in both eyes (black arrowheads). **(k,l)** OCT scan, showing loss of RPE in fovea and perifoveal region, epiretinal membrane and atrophic plaques in central macula. The right pictures of central macula evidence a large plaque of atrophy in OD fovea and four smaller parafoveal plaques in OS (black arrowheads). Red and yellow arrowheads indicate start and end position, respectively for atrophic RPE loss in fovea (OD). **(m–p)** Fundus imaging of the right and left eye of the individual II:8 from family RP-0476 at 55 years-old. (**m,n**) Fundus imaging, revealing macular RPE alteration, round and bone-spicule pigmentation in mid-periphery.(o-p) FAF, showing perifoveal hypoautofluorescence ring **(q,r)**. OCT of both eyes, revealing conservation of inner and outer segments in fovea, epiretinal membrane and atrophy of external layers in parafoveal area. Blue arrowheads indicate the localization of preserved RPE in central fovea.

The clinical course and visual outcome of the 5 patients carrying the p.Arg630* mutation in *SAMD11* were reviewed, as detailed in Table 1. Consistently, patients were diagnosed of RP between the third and fourth decade of life, presenting night blindness as first symptom and followed by progressive constriction of visual field. Overall, patients have a central visual field restricted between <10° and absolute scotoma. Loss of visual acuity was also observed in late stages of the disease, being the best-corrected visual acuity (BCVA) between 20/32 and hand movement, except for the youngest patient (II:8, Family RP-0476) who still maintained a well conserved BCVA at 55 years old (Table 1). When available, ERG registers were non-recordable in both scotopic and photophic conditions. Funduscopies showed typical RP changes as pale papilla, narrowed retinal vessels, abundant pigmentary changes in mid periphery and retinal pigment epithelium (RPE) atrophy in mid-periphery and in fovea (Fig. 2). Interestingly, similar findings on central retina were observed in two patients from different families, consisting in large plaques of atrophy, as revealed by optical coherence tomography (OCT) and fundus autofluorescence images (individual II:7, Family RP-1105: Fig. 2a,c and e; individual II:8, Family RP-0476: Fig. 2g–l). Macular OCTs also confirmed a generalized degeneration of rods, being compatible with diagnosis of RP, while cones were preserved only in fovea (Fig. 2). Bilateral posterior subcapsular cataracts were also present at both eyes in all patients.

### SAMD11 transcripts are widely expressed in human retina and extra-ocular tissues

The human *SAMD11* reference transcript harbours 14 exons and encodes a 681 aa protein (Fig. 1c) that belongs to the SAM protein superfamily, characterized by the presence of the evolutionarily conserved sterile alpha motif (SAM) domain. A recent cloning of the human *SAMD11* allowed the identification of up to 45 alternative splice variants^21^. Several alternative N-*termini* were described; however, all the isoforms that expect to be translated into proteins share the same C-terminal part. We investigated the expression profile in different human tissues by RT-PCR experiments using specific primers for the well-conserved 3′ region of *SAMD11*. We found expression of *SAMD11* in all the 22 of the tissues tested except in whole blood (Fig. 1d). In concordance, SAMD11 could not be detected in lymphoblastoid cell lines derived from several control individuals and a homozygous carrier of the p.Arg630* mutation (Supplementary Figure S4). Thus, *SAMD11* is widely expressed, showing the highest expression in kidney, prostate and human retina.

### Immunolocalization of SAMD11 in human retina

**Figure 3:**
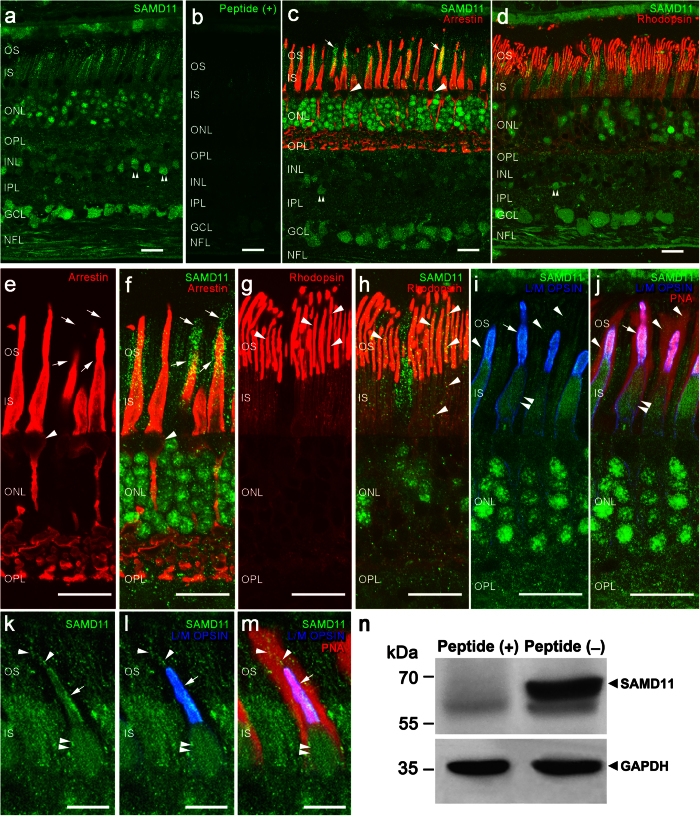
Immunolocalization of SAMD11 in vertical sections of human retina. **(a)** SAMD11 is located in rod photoreceptor cell bodies in the ONL and in the inner and outer segments of cones and rods. A subset of amacrine cells (double arrowheads in a, c, d) also exhibits expression of SAMD11 protein. Besides, ganglion cells evidenced SAMD11 immunolabeling in their cell bodies and axons, which constitute the nerve fiber layer. **(b)** Pre-absorption control for SAMD11 antibodies specificity using the immunogenic peptide. **(c,e,f)** Double immunostaining with antibodies against SAMD11 and cone arrestin, a specific marker for cone photoreceptors, showed immunoreactivity of SAMD11 in the inner and outer segments of cones andextracellular matrix (arrows). **(d,g,h)** SAMD11 and rhodopsin, a specific marker that labels rod outer segments, double immunolabeling showed the localization of SAMD11 in rod inner and outer segments (arrowheads). **(I,j)** Long/medium wavelength opsin antibodies and peanut agglutinin lectin staining evidenced the presence of SAMD11 in outer and inner segments of cones, as well as in their extracellular matrix. **(k–m)** Detail of the immunolocalization of SAMD11 in cone outer segments and extracellular matrix. **(n)** Immunoblotting analysis of SAMD11 protein. Human retina samples expressed strongly immunoreactive bands detected with SAMD11 or GAPDH antibodies. The arrowheads point to the 68 and 36 kDa protein bands corresponding to SAMD11 and GAPDH, respectively. Protein molecular weight markers are given to the left. SAMD11 immunolabeling (peptide−) was specifically abolished when the SAMD11 antibody was preincubated with its immunogen peptide (peptide+). (**a–h)** represent a confocal stack projection of 5 pictures and (**i–m)** are single confocal images. Sky-blue color observed in (**i**,**l)** is the result of the co-localization in the outer segments of red/green cones of the immunoreactivity for long/medium wavelength opsins (dark-blue color) and SAMD11 (green color). OS, outer segments; IS, inner segments; ONL, outer nuclear layer; OPL, outer plexiform layer; INL, inner nuclear layer; IPL, inner plexiform layer; GCL, ganglion cell layer; NFL, nerve fiber layer. Scale bar a-i = 20 μm;k–m = 10 μm.

To shed light on the implication of SAMD11 in retinal physiology, we investigated its expression and localization pattern in the distinct retinal cell types on adult healthy human retina by means of Western blotting and confocal immunofluorescence microscopy (Fig. 3). Immunoblotting analysis revealed the presence in the mentioned tissue of a prominent and specific immunoreactive band with apparent molecular weight of 68 kDa corresponding to the SAMD11 protein (Fig. 3n).

On the other hand, we characterized the SAMD11 distribution pattern in cryo-fixed vertical sections of human retina, which were immunolabeled with specific SAMD11 antibodies. As a result, SAMD11 protein was found in the three nuclear layers of the retina: outer and inner nuclear layers (ONL and INL, respectively), and ganglion cell layer (GCL) (Fig. 3a,c–d). Specifically, SAMD11 immunoreactivity was observed in a small population of amacrine cells located in the INL (Fig. 3a,c,d; double arrowheads), as well as in most of ganglion cells and their axons in the nerve fiber layer (NFL). SAMD11 was also detected in photoreceptors cells and interestingly we observed differential expression of this protein between cone and rods. In this regard, SAMD11 immunoreactivity was not detected in cone cell bodies (Fig. 3c,e,f; arrowheads) whereas rod cell bodies evidenced a prominent SAMD11 expression (Fig. 3a,c,d,f,h–j). Double immunolabelling of SAMD11 with cone arrestin, a specific marker for cone photoreceptors, revealed that SAMD11 protein was present in the inner and outer segments of cones (Fig. 3c,e,f), including the extracellular matrix (Fig. 3c,e,f; arrows). We double-checked the presence of SAMD11 in the outer segments of cones combining the SAMD11 antibody with long/medium wavelength opsin antibodies, which are specific markers for the outer segments of red/green cones (Fig. 3i–l). Double labelling with antibodies anti-SAMD11 and the peanut agglutinin lectin showed the localization of SAMD11 in the extracellular matrix of cones (Fig. 3i–m; arrowheads). Similarly to cones, SAMD11 immunoreactivity was observed in rod inner and outer segments (Fig. 3e,f), as verified the co-localization of this protein with rhodopsin, a specific marker that labels rod outer segments (Fig. 3d,g,h; arrowheads). Furthermore, Fig. 3i–k showed clearly the presence of SAMD11 in the outer (Fig. 3j,k; arrows) and inner segments (ellipsoid) of cones (double arrowheads), as well as in the extracellular matrix of cones (Fig. 3i–m; arrowheads). No immunoreactivity was found against SAMD11 in retina using the preabsortion of the antibody with their specific peptide (Fig. 3b).

## Discussion

In the present study, we report a novel homozygous nonsense mutation in *SAMD11*, which was identified using homozygosity mapping followed by exome sequencing. Our findings provide evidence for the first association of this gene with an inherited retinal dystrophy. Five patients with late-onset Retinitis Pigmentosa from two unrelated families carried this mutation homozygously. In addition, after *SAMD11* screening in our cohort, another three novel very likely pathogenic variants were also identified in heterozygous state. In these heterozygous patients, a second allele in coding region or large CNVs were discarded, however, we cannot exclude the presence of a second pathogenic variant in regulatory or deep intronic regions.

SAMD11 is a highly conserved protein from zebrafish to human, and has an isolated SAM domain in their C-terminus, without another known motif (Fig. 1c). SAMD11 was first described as a predominantly expressed protein in the terminal stage of photoreceptor differentiation^11^. In developing mouse retina, Samd11 expression begins at E18 with a peak level at P6^11^, when rod outer segments formation occurs^22^. In adult humans, SAMD11 mRNA and protein expression have been determined in different ocular tissues, including the retina^21^,^23^. Consistent with these previous studies, our gene expression analysis showed that *SAMD11* is a widely expressed gene, being present in both ocular and extra-ocular tissues. Additionally, among them, we detected higher values of SAMD11 expression in the retina. Moreover, in the present study, we identified for the first time the neural localization pattern of SAMD11 in the human retina by immunohistochemistry. As a consequence, SAMD11 was mainly localized in ONL, where the rod photoreceptor cell bodies are located, as well as in a small population of amacrine cells located in INL and in most of ganglion cells and their axons in NFL. The prominent SAMD11 immunoreactivity observed in rod cell bodies is indicative of a relevant SAMD11 role for the correct function of rod photoreceptors in the adult human retina. Hence, dysfunction of this protein could be critically involved in the primary rod loss underlying the RP pathogenesis.

The specific localization in human retina and its specific temporal prenatal and postnatal expression pattern in mouse correlating with developing and maturing of rod^11^,^22^ suggest a potential role of SAMD11 in photoreceptor differentiation and survival. Early fate and terminal differentiation of rods are mainly controlled by a hierarchical regulatory network including several transcription factors (TF), such CRX, the orthodenticle homeobox 2 (OTX2), neural retinal leucine (NRL) and the orphan nuclear receptor NR2E3^24^,^25^,^26^,^27^,^28^. Interestingly, all of them have been involved in the rod dysfunction underlying retinal dystrophies^29^,^30^,^31^. As occurs in most of human genes associated with retinal dystrophies^32^, the retinal expression of *SAMD11* seems to be directly regulated by CRX and OTX2 through several highly conserved binding sequences in the promoter region, as supported by different *in vitro* and *in vivo* studies^11^,^21^,^33^. Recently, several RP-associated genes, such as *FAM161* and *MAK,* have been identified as candidate genes using the mouse retinal CRX targetome obtained by ChIP-seq^34^,^35^. Thus, prioritization of CRX target genes have revealed as a very effective strategy to pinpoint novel candidate retina-specific genes. Remarkably, in this experiment, the principal CRX-target is *SAMD7*, the closest phylogenetic relative of *SAMD11* (Supplementary Figure S5) with a very similar expression profile in the human and mouse retina^32^,^36^. Although *SAMD11* was apparently not included as a potential CRX-target in the above ChIP-seq dataset, we noticed that the mouse genome assembly (mm9) used at that time did not include yet the *Samd11* gene. After converting genome coordinates to the most actualized assembly (mm10), we found that between the 100 most enriched CRX-bound regions (CBRs) identified by Corbo and collaborators, there was one CBR located to the promoter of mouse *Samd11* (Supplementary Figure S6)^32^. This CRB seems to be actively transcribed by RNA Polymerase II complexes at mouse developing neural retina^37^, suggesting that these regulatory regions can act as initiation and elongation sites of *SAMD11* transcription.

SAM domains are involved in protein-protein interactions during signal transduction and transcriptional regulation^38^,^39^. SAM domains, that are arranged in a small 4–5-helix bundle of two orthogonally packed α-hairpins^40^ (Supplementary Figure S5), can homo- and hetero-oligomerise, forming multiple self-association architectures^41^. In this sense, it was described the mouse Samd11 protein is able to self-associated mainly through the SAM domain^11^. SAM proteins have been implicated both in normal and pathological processes of eye development. In *Drosophila* eyes, Yan, Mae and Pointed-P2 are SAM domain-containing proteins acting as transcriptional factors of the Ras-MAPK pathway^39^,^42^,^43^. In these proteins, SAM domain plays an important role in the transcriptional activity via heterotypic interactions, as suggested by *in vitro* studies^44^,^45^. It is unknown whether comparable SAM-mediated interactions could influence photoreceptor development in Mammals. A SAM domain is also found in the SANS/USH1G protein, a scaffolding protein involved in the pathogenesis of the Usher syndrome type 1^46^.

Experimental evidences suggested that *SAMD11*, similarly to *SAMD7*, is implicated into the CRX-mediated transcription acting as transcriptional repressor^11^,^36^. Its interaction with yet-unknown proteins could promote rod fate and/or maintenance. It is noteworthy that this transcriptional regulation seems to be exerted without the presence of an obvious DNA binding domain. In addition, repressor activity of SAMD11 is not due to SAM interactions but it resides at the conserved C-terminal region^11^. Remarkably, it is the C-terminus domain, but not the SAM domain, which is lost in the homozygous RP patients carrying the truncating mutation p.Arg630*, evidencing a likely important role of this domain. In an effort to provide more experimental evidence of the involvement of the mutation identified in this work, LCLs derived from a homozygous carrier were obtained and additional experiments of *SAMD11* expression were performed, comparing with control individuals. Unfortunately, we could not detected neither RNA nor protein expression in LCLs (Supplementary Figure S4). Thus, the specific functional role of SAMD11 remains unclear and warrants further research.

Identification of *SAMD11* as causative gene in two RP families highlights a putatively important role for other SAM-related proteins, such *SAMD7,* in the pathogenesis of the retinal dystrophies. *SAMD11* and *SAMD7* share common features including their site and timeline of expression in the mouse retina, their nuclear localization, the presence of a very similar C-terminal SAM motif, lack of additional functional domains, their regulation by CRX and a very likely function as transcriptional repressors. In view of their high expression levels in the retina, and the weak expression of other SAM family members with isolated SAM domain^36^, it has been suggested a likely interaction of both proteins in the retina. Therefore, it could be very interesting to determine if they can act synergistically or if they have overlapping functions in the retina. Supporting this last supposition, the patients carrying the deleterious nonsense mutation in *SAMD11* developed a late rod affectation with first symptoms of night blindness and field constriction in the third to fourth decade of life. By contrast, mutations in *CRX* and other CRX-regulated genes, such *RS1, FAM161* and *MAK*, are responsible of congenital and early-onset forms of retinal dystrophy^29^,^34^,^35^,^47^,^48^.

Recently, a very rare missense variant in *SAMD11* has been putatively associated with autism spectrum disorders (ASDs)^49^, suggesting that *SAMD11* could be a good candidate for autism. However, none of patients carrying the novel mutations and variants in *SAMD11* here reported suffer from autistic behaviour, related-neurodevelopmental disorders or intellectual disability. By contrast, we report a very homogeneous phenotype in both families consisting in Retinitis Pigmentosa with atrophic macular RPE degeneration in late stages of the disease. Significant and distinctive plaques of atrophy were clearly observed in late stages of disease in FAF and OCT.

In brief, we have identified a nonsense mutation in a novel gene as cause of adult-onset RP in five patients. The identification of a *SAMD11* truncating mutation affecting the C-terminus of the protein highlights the putative importance of this domain both in the repressive function of this gene and in RP pathogenesis. Our findings strongly suggest the involvement of this protein in the development of the rod degeneration in human and in photoreceptor maintenance. This work contributes to shed further light on the molecular mechanisms underlying the pathogenesis of the retinal dystrophies. Further research on *SAMD11* is expected to provide insights into its specific role in the retina and its pathogenic mechanism responsible for Retinitis Pigmentosa.

## Materials and Methods

### Subject recruitment and clinical evaluation

A total of 560 unrelated Spanish families with different IRDs are included in this study: 486 families with arRP and 74 families with Leber congenital amaurosis (LCA). In addition, 196 Spanish healthy unrelated individuals were used as a control samples. They were randomly selected from blood donors who voluntarily participate in this study after filling out a questionnaire specifically designed to inquire about ophthalmic diseases. They did not report any personal or familial history of retinal dystrophy. All patients and control individuals were collected at Fundación Jiménez Díaz University Hospital (FJD, Madrid, Spain). Written informed consent was obtained from all subjects or their legal guardians prior to their participation in this study, also covering the publication of the clinical data. All procedures were approved by the FJD Ethics Committee and adhered to the tenets of the Declaration of Helsinki.

Diagnosis and follow-up of patients were based on ophthalmic evaluation including measurements of BCVA and visual field tests, fundus and OCT examination and ERG responses. Diagnostic criteria of LCA included severely impaired bilateral visual function at birth or before one year-old, congenital nystagmus, weak pupillary responses and non-detectable or severely reduced ERG. Diagnostic criteria of RP included poor night vision and/or peripheral visual, with poor visual acuity and visual field loss in advanced stages of the pathology.

Genomic DNA was obtained from peripheral blood samples using an automated DNA extractor (BioRobot EZ1 Qiagen, Hilden, Germany) following the manufacturer instructions. Known mutations in LCA or ARRP genes were previously excluded in index cases using a genotyping microarray based on Arrayed Primer Extension (APEX) technology (LCA or ARRP chip, AsperOphthalmics, Tartu, Estonia). In addition, for families RP-1105 and RP-0476, direct Sanger sequencing of the coding exons and flanking intronic sequences of the *EYS* gene^50^ and *ABHD12 and ZNF408,* two new genes recently associated to arRP by our group^9^,^10^, was performed and no pathogenic variants were found.

Lymphoblastoid cell lines (LCLs) were established by Epstein Barr virus (EBV)-transformation of peripheral blood lymphocytes from one patient (II:7, family RP-1105) and three control individuals. Generation of EBV-derived LCLs was performed by the CIBERER Biobank (Valencia, Spain). Cell lines were cultured in RPMI-1640 media (GIBCO/BRL, Grand Island, NY, USA) supplemented with 10% fetal bovine serum (Gibco), 1% (v/v) antibiotics/antimycotics that included penicillin-streptomycin (Gibco) and 2 μg/ml Fungizone (Gibco). The cultures were carried out in 25 cm^2^ flasks at 37 °C in 5% CO_2_ atmosphere.

### Homozygosity mapping

Whole-genome homozygosity mapping was performed using high-resolution commercial SNP arrays from Illumina (HumanCytoSNP-12 SNP microarrays, Illumina, San Diego, CA, USA). Arrays were processed according to the manufacturer′s protocols. IBD regions were calculated as previously reported^51^,^52^. Regions of homozygosity were interrogated for the presence of retinal disease-associated and candidate genes were screened by Sanger sequencing.

### Whole-exome sequencing

Whole-exome sequencing analysis was performed by the Spanish Centre for Genome Analysis (CNAG, Barcelona, Spain). Genomic DNA was enriched for exonic sequences of approximately 30,000 genes using the Agilent SureSelect Human All Exon version 4 kit (Agilent Technologies, Santa Clara, CA, USA), following manufacturer’s standard protocol. Captured DNA library was sequenced on a HiSeq 2000 sequencing platform (Illumina) to generate pair-end reads up to 100 cycles.

Base calling and quality control were performed using the Illumina RTA sequence analysis pipeline. Analysis of the primary data (FASTQ files) was done using the BIER´s platform pipeline (BiERapp: http://bierapp.babelomics.org/)^10^. Sequence reads were aligned to the human reference genome build GRCh37 (hg19) using the Burrows-Wheeler Aligner (BWA)^53^. Mapped reads were filtered (leaving only those mapping in unique genomic positions with enough quality), sorted and indexed with SAMtools. GATK was then used to realign the reads as well as for the base quality score recalibration^54^. Once a satisfactory alignment was achieved, identification of SNVs and INDELs was performed using GATK standard hard filtering parameters^55^. For the final exome sequencing analysis report we used the VARIANT annotation tool, which provide additional relevant variant information for the final process of candidate gene selection^56^. In particular, MAF, obtained from dbSNP database, 1000 Genomes^14^ and EVS^15^ projects, was provided to help on the selection of new variants not reported in healthy population to date^14^,^57^. SIFT and Polyphen damage scores were computed to predict the putative impact of the discovered variants over the protein structure and functionality^58^,^59^. This information was completed with the evolutionary conservation obtained from PhastCons^60^. Also disease related annotations were provided at both variant and gene levels if available. Finally, GO terms for the affected genes were also retrieved. The successive application of quality control filters and the prioritization by the parameters accounting for potential functional impact led us to build up a list of candidate genes (and variants) ranked by its segregation with the cases and the putative potential impact. Such prioritization list was further inspected to look for potential candidate genes and/or variants.

### Targeted NGS

A customized panel including two candidate genes (*SAMD11* and *ZNF408*^18^,^19^) and 73 previously known RD genes was developed using the Haloplex capture technology (Agilent Technologies Inc., Santa Clara, CA, USA), as previously described^18^,^19^. Amplicons for all coding and non-coding exons, including 20 bp of flanking 5′ and 3′ intronic sequence were designed using the SureDesign webtool (Agilent Technologies, https://earray.chem.agilent.com/suredesign/).

Using this approach, the 14 target regions of *SAMD11* were completely covered with 123 amplicons. Target enrichment was performed according to HaloPlex Enrichment System (Agilent Technologies) for Illumina Sequencing protocol with some modifications previously described^18^,^19^. Captured target libraries from 180 probands were sequenced on an Illumina MiSeq system (11 samples per run) to obtain 150 bp paired-end reads. A specific custom pipeline for HaloPlex kits on Illumina implemented into the commercial DNAnexus platform (https://www.dnanexus.com/) was used for the bioinformatic analysis, as previously described^18^,^19^.

### Mutational screening

Bidirectional automatic sequencing was performed in order to confirm and segregate the obtained results by NGS, to determine the frequency of novel variants in a cohort of 380 autosomal recessive or sporadic RP patients and a control cohort and, also to screen the *SAMD11* gene in additional 220 patients diagnosed with adulthood - onset arRP. Primers for amplification of the coding exons and splice boundaries of *SAMD11* were specifically designed using Primer3 software (Supplementary Table S6). PCR products were enzymatically purified with ExoSAP-it (USB, Affymetrix, Santa Clara, CA, USA) and sequenced on both strands using Big Dye Terminator Cycle Sequencing Kit v3.1 Kit (Applied Biosystems, Waltham, MA, USA). The PCR products were purified on a 96-well multiscreen filter plate (Montage SEQ96 Sequencing Reaction Cleanup Kit, Millipore, Bedford, MA) and resolved on an automated sequencer (ABI 3130xl Genetic Analyzer, Applied Biosystems).

### Haplotype analysis

For the STRs genotyping, PCR products were electrophoresed using the automated ABI 3130xl Genetic Analyzer (Applied Biosystems) and analyzed with the GeneMapper v3.5 software (Applied Biosystems). Polymorphic microsatellites with high heterozygosity located at telomeric region of short arm of chromosome 1 were searched on public databases. Haplotype reconstruction was performed using the software Cyrillic ver. 2.1 (Cyrillic Software, Wallingford, UK).

### Assessment of the pathogenicity of new variants

The pathogenicity of unreported variants was established by the following criteria: i) co-segregation in the family, ii) absence in 196 Spanish healthy control individuals after screening by Sanger sequencing and in variant databases, such as 1000 genomes^14^ and EVS^15^ (http://evs.gs.washington.edu/EVS/), and the CIBERER Spanish Variant Server (http://csvs.babelomics.org/), iii) amino acid conservation for missense mutations in orthologs of the SAMD11 belonging to different evolutionary branches, and iv) pathogenicity prediction with *in silico* tools, such as Align-GVGD (http://agvgd.iarc.fr/agvgd_input.php)^61^, SIFT (http://sift.jcvi.org/)^58^, PolyPhen-2 (http://genetics.bwh.harvard.edu/pph2/)^59^, Protein Variation Effect Analyzer (PROVEAN; http://provean.jcvi.org/index.php)^62^, and Human Splicing Finder (HSF; http://www.umd.be/HSF/)^63^. The BLINK tool and the Jalview Alignment Editor program were used to analyze the multiple sequence alignments.

### Array-based comparative genomic hybridization (aCGH)

A custom aCGH 8×60k using the Agilent SurePrint G3 CGH technology was designed using the Agilent eArray website (https://earray.chem.agilent.com/earray/) with an average distribution of 1 probe per 150 bp in the *SAMD11* gene with a total of 95 probes. Briefly, genomic DNA (200 ng) from the patient and from a sex-matched control were digested by *Alu*I and *Rsa*I restriction enzymes for 2 h at 37 °C and the digested products were labelled with Cy3-dUTP and Cy5-dUTP fluorochromes using the Sure Tag DNA Labeling Kit (Agilent Technologies). The labelled products were purified, hybridized and washed according to Agilent protocols. The slide was scanned on a SureScan G4900DA scanner (Agilent Technologies), and the resulting TIFF images were converted by the image conversion Feature Extraction software (Agilent Technologies). Results were analyzed by Agilent CytoGenomics software v.2.7 using default analysis method – CGH v2 with the ADM-2 aberration algorithm.

### Reverse transcription PCR (RT-PCR)

Blood RNA was isolated from peripheral blood lymphocytes using PAXgene blood RNA kit (Qiagen), according to the protocol provided by the manufacturer. A commercial panel of human RNA from 22 different human tissues (Human Total RNA Master Panel II, Takara Bio Clontech, CA, USA) and human retina (Human Retina QUICK-Clone™ cDNA, Takara Bio Clontech) were analysed by RT-PCR. Total RNAs were reversely transcribed to cDNA with ImProm-II™ Reverse Transcription System (Promega, Madison, WI, USA) using random primers. RT-PCR experiments were performed using *SAMD11* exonic primers pairs spanning exons 12, 13 and 14 (Supplementary Table S6). Primers for the housekeeping *GAPDH* gene were used as internal control. Subsequent Sanger sequencing of the RT-PCR products confirmed correct *SAMD11* amplification.

#### Immunohistochemistry

Anonymized human retina samples were obtained from the eye donors that were collected and stored at the Eye BioBank from Hospital General Universitario de Alicante (Alicante, Spain). Written informed consents were obtained from relatives who donated voluntarily eyeballs for use in research procedures. All experiments were performed in accordance with relevant guidelines and regulations. All procedures were approved by the Ethics Committee from University of Alicante and adhered to the tenets of the Declaration of Helsinki.

Eyes were fixed in 4% paraformaldehyde for 2 hours at room temperature and after washing, cryoprotected using a sucrose gradient. Vertical sections of 16 μm thickness were cut on a cryostat and were immunostained at room temperature overnight with goat polyclonal antibodies to human SAMD11 from Santa Cruz Biotechnology (Santa Cruz, CA, USA; Catalog No. sc-248525) at a 1:50 dilution in 0.1 M sodium phosphate buffer (pH 7.4), 0.5% Triton X-100 in the presence or absence of blocking peptide (20:1 peptide:antibody ratio; Santa Cruz; Catalog No. sc-248525P). Double immunohistochemistry were performed using SAMD11 antibodies in combination with monoclonal mouse anti-rhodopsin at a 1:500 dilution (Millipore Temecula, CA, USA; Catalog No. MAB5356) or monoclonal mouse anti-cone arrestin at a 1:200 dilution (Dr. MacLeish, Morehouse School of Medicine; Atlanta, GA, USA)^64^, or polyclonal rabbit antibodies anti-red/green opsins at a 1:200 (Millipore; Catalog No.AB5405). Subsequently, the sections were incubated at room temperature for 1 h with Alexa Fluor 488 donkey anti-goat IgG or Alexa Fluor 546 donkey anti-goat IgG, Alexa Fluor 555 donkey anti-mouse IgG and/or Alexa Fluor 633 donkey anti-rabbit IgG secondary antibodies from Molecular Probes (Eugene, OR, USA) at a 1:100 dilution. FITC-labeled peanut agglutinin (PNA) (Vector laboratories, Peterborough, UK; Catalog No.FL-1071) at a 1:100 dilution was incubated together with secondary antibodies. Images were finally obtained under a Leica (Wetzlar, Germany) TCS SP2 confocal laser-scanning microscope.

#### Western blotting

SAMD11 protein expression was assessed using Western blotting on adult healthy human retina. Proteins (40 μg/lane) were resolved by SDS-PAGE on 4–20% polyacrylamide-gradient gels and electrotransferred to Hybond-P PVDF membranes (GE Healthcare, Buckinghamshire, UK). These were probed at 4 °C overnight with the same SAMD11 antibodies used in immunohistochemistry assays at a 1:500 dilution in 25 mM Tris (pH 8.0), 150 mM NaCl, 2.7 mM KCl (TBS) in the presence or absence of blocking peptide (10:1 peptide:antibody ratio), or with mouse monoclonal antibodies to rabbit muscle GAPDH at a 1:1,000 dilution (Millipore; Catalog No. MAB374). Thereafter, the membranes were incubated at room temperature for 1 h with horseradish peroxidase-conjugated donkey anti-goat (Abcam, Cambridge, UK) or goat anti-mouse (Pierce, Rockford, IL, USA) IgG at a 1:20,000 dilution. Detection was performed by enhanced chemiluminescence using the SuperSignal West Dura system (Pierce).

### Network analysis

Network analysis of the candidate gene products was carried out with the SNOW tool, implemented in the Babelomics web package (http://www.babelomics.org/)^16^,^65^. SNOW identifies the proteins corresponding to the candidate genes within the human interactome, calculates the Minimal Connected Network (MCN) (the smallest network that connects all the genes in the list) allowing one intermediate interaction and, finally, evaluates its topology by comparing the average clustering coefficient of the MCN versus the resulting value of this parameter in empirical MCNs generated from 1000 random gene lists of same size. The clustering coefficient accounts for the propensity of proteins in the MCN to form a connected unit.

## Additional Information

**How to cite this article**: Corton, M. *et al*. Identification of the Photoreceptor Transcriptional Co-Repressor *SAMD11* as Novel Cause of Autosomal Recessive Retinitis Pigmentosa. *Sci. Rep.* **6**, 35370; doi: 10.1038/srep35370 (2016).

## Supplementary Material

Supplementary Information

## Figures and Tables

**Table 1:** Clinical features of the 5 patients carrying the mutation p.Arg630* in the *SAMD11* gene.

Family	Patient ID	First symptoms and course	Age at diagnosis (y)	Age of Ophthalmic Evaluation (y)	BCVA OD/OS	VF OD/OS	ERG	Fundus aspect	OCT		Additional findings
									Macula	Optic nerve	
RP-1105	II:5	NB, field constriction and progressive loss of VA	NA	75	0.2/0.2	<8°/<8°	NA	Pale optic disc, narrowed vessels, bone spicule pigmentation in mid periphery	NA	N	Subcapsular cataracts
	II:6	NB, field constriction and progressive loss of VA	NA	63	0.6/0.6	<10°/<10°	NR	Normal optic disc, narrowed vessels and bone spicule pigmentation in mid periphery	Macular oedema	NA	Subcapsular cataracts
	II:7	NB (36y), field constriction (53y) and progressive loss of VA (60y)	38	66	0.6/0.5	Absolute scotome (BE)	NR	Pale optic disc, retina vessels attenuation, round and bone spicule pigmentation and RPE atrophy at both eyes. Atrophic plaque at macula (OD). Vitreous veils	OD: Atrophy of external layers of retina with RPE loss in fovea. OS: inner and outer segment conservation in fovea. Epiretinal membrane (BE)	N	Photophobia, dyschromatopsia and nuclear and posterior subcapsular cataracts
RP-0476	II:4	NB (37y), field constriction (50y) and progressive loss of VA (60y)	38	74	HM/0.05	<10°/<10°	NR	Pale optic disc, retina vessels attenuation and moderate bone spicules pigmentation in mid and extreme periphery. RPE alteration in BE. Central atrophic plaque in OD and several small atrophic plaques in OS.	Atrophic plaques in fovea and parafoveal area, absence of RPE in fovea. Epiretinal membrane	NA	Nuclear and posterior subcapsular cataracts. Cornea *guttata*. Myopia
	II:8	NB and field constriction	28	55	1/0.8	<10°/<5 °C	NR	Mild pale optic disc, narrowed vessels, and abundant round and osteoclast pigmentation in mid-periphery, RPE alteration in mid-periphery and macula.	Inner and outer segment preservation in fovea, with atrophy of external layers in perifoveal region. Epiretinal membrane.	N	Pseudophakia and posterior capsulotomy BE, nasal pterigium in OD, hyperopia and astigmatism

BCVA, best corrected visual acuity; BE, both eyes; ERG, electroretinogram; HM, hand movement; ID, identification; N, normal; NA, no available; NB, night blindness; NR, non recordable; OCT, optical coherence tomography; OD, right eye; OS, left eye; RPE, retinal pigment epithelium; VA, visual acuity; VF, visual field; y, years.
