# Supplementary material for: Identification of the Photoreceptor Transcriptional Co-Repressor SAMD11 as Novel Cause of Autosomal Recessive Retinitis Pigmentosa
Source: Sci Rep. 2016 Oct 13;6:35370. doi: 10.1038/srep35370 (PMC5062157; doi:10.1038/srep35370)
Supplement: Supplementary Information [file srep35370-s1.pdf]

Identification of the Photoreceptor Transcriptional Co-Repressor *SAMD11* as Novel Cause of Autosomal Recessive Retinitis Pigmentosa

Corton M[1,2]*, Avila-Fernández A[1,2] , Campello L[3], Sánchez M[1,2], Benavides B[1,2], López-Molina MI[4], Fernández-Sánchez L[3], Sánchez-Alcudia R[1,2], da Silva LRJ[1,2,5],Reyes N[1,2], Martín-Garrido E[1,2], Zurita O[1,2], Fernández-San José P[1,2], Pérez-Carro R[1,2], García-García F[6,7], Dopazo J[6,7,8], García-Sandoval B[4], Cuenca N[3], Ayuso C[1,2]*

[1] Department of Genetics & Genomics, Health Research Institute–Jiménez Díaz Foundation University Hospital (IIS-FJD), Madrid, Spain.

[2] Centre for Biomedical Network Research on Rare Diseases (CIBERER), ISCIII, Madrid, Spain.

[3] Department of Physiology, Genetics and Microbiology, University of Alicante, Alicante, Spain.

[4] Department of Ophthalmology, Health Research Institute– Jiménez Díaz Foundation University Hospital (IIS-FJD), Madrid, Spain.

[5] Universidade de Mogi das Cruzes, São Paulo, Brazil.

[6] Computational Genomics Department, Centro de Investigación Príncipe Felipe (CIPF), Valencia, Spain.

[7] Bioinformatics in Rare Diseases (BIER), Centre for Biomedical Network Research on Rare Diseases (CIBERER), Valencia, Spain.

[8]Functional Genomics Node (INB), Valencia, Spain

* Correspondence: Carmen Ayuso: cayuso@fjd.es

Marta Corton: mcorton@fjd.es

Ranking	Chromosome	Start position	End position	Size (Mb)	No. Genes	Retinal Disease- genes
1	3	150,692,298	171,108,599	20.42	176	*CLRN1*[a]
2	1	18,4217,979	196,099,926	11.88	57	
3	1	752,566	2,026,749	1.27	69	

Supplementary Table S1. Overview of the shared homozygous regions found by high-resolution single nucleotide polymorphism (SNP) arrays in the three siblings of the family RP-1105. Homozygosity regions larger than 1 Mb are shown. Chromosome position based on human genome hg19 built, the size of the region, total number of genes within each giving chromosomal interval and known genes associated to retinal dystrophies are shown.[a] Mutations were excluded by Sanger sequencing.

WES findings	No. Variants
**Total unique mapped variants**	**8,127**
**Potentially functional variants**	**7,525**
Heterozygous	3,754
Homozygous	3,771
Non-synonymous SNVs	7,099
Splicing	56
Stopgain	59
Stoploss	26
Frameshift indel	193
Non-frameshift indel	92
**Variants MAF ≤ 0.5%**	**296**
Heterozygous	222
Homozygous	74
**Variants within LOH regions**	**2**
Non-synonymous SNVs	1
Stopgain	1

Supplementary Table S2. Overview of the whole-exome sequencing (WES) findings in the proband of the family RP-1105. Filtering was based on the following criteria: 1) Variants in coding regions of target captured protein coding and potentially functional, i.e. non-synonymous, splice acceptor and donor site single nucleotide variations (SNVs), frameshift and non-frameshift indels; 2) Novel or rare: with a minor allele frequency (MAF) ≤ 0.5% in dbSNP, 1000 genomes and EVS databases; 3) Autosomal-recessive model and 4) Variants within the shared regions of homozygosity previously found by genome-wide homozygosity mapping.

Genomic position (hg19)	Rank IBD	Gene	Nucleotide variation	Amino-acid variation	Mutation type	MAF	SIFT prediction (score)	Polyphen prediction (score)	Segregation
1:879,375-879,375	3	***SAMD11***	NM_152486.2:c.1888C>T	p.Arg630*	Stopgain	N.D	---	----	Yes
1:907,797-907,797	3	***PLEKHN1***	NM_032129.2:c.995G>A	p.Gly332Glu	Missense	N.D	Deleterious (0.04)	Damaging (0.949)	Yes

Supplementary Table S3. Novel or rare variants found by exome sequencing within the previously known regions of homozygosity in the family RP-1105. Two novel variants were found in the 3th shared region of homozygosity of 1.27 Mb. None of them were described at dbSNP. 1000 genomes or EVS databases. Both variants were further confirmed by Sanger sequencing and segregated correctly in the family. The amino acid substitution is predicted damaging by if the SIFT score is <= 0.05. Polyphen predicts a non-synonymous variant as probably damaging if score is > 0.85. MAF: Minor allele frequency. SNV: Single Nucleotide Variation. N.D.: Not described at databases.

Exon	Nucleotide change	Amino-acid variation	Status	ID	MAF (1K genomes)	No. Alleles
Nonsense						
6	**c.502C>T**	**p.Arg168***	HET	N.D.	N.D.	1
14	**c.1888C>T**	**p.Arg630***	HOM	N.D.	N.D.	4
Splicing						
14	**c.1801-2A>C**		HET	N.D.	N.D.	1
Missense						
3	**c.133A>G**	**p.Lys45Glu**	HET	N.D.	N.D.	1

Supplementary Table S4. List of novel *SAMD11 variants* identified in this study. Nucleotide numbering is based on RefSeq DNA accession number NM_152486.2. ID: Identification in the dbSNP database. MAF: Minor allele frequency, considering the 1000 genomes database. N.D.: Not described.

Nucleotide variation	Amino-acid variation	Conservation	Mutation Taster	PROVEAN	PolyPhen	SIFT	Remarks
c.133A>G	p.Lys45Glu	HC	Disease causing (56)	Deleterious (-2.630)	Probably damaging (0.999)	Affect protein function (0.01)	Not found in 384 control Spanish chromosomes.

Supplementary Table S5. *In silico* predictions of the novel non-synonymous *SAMD11* variant. HC: Highly conserved, considering 30 orthologs of the *SAMD11* protein belonging to different evolutionary branches. The amino acid substitution is predicted damaging if the SIFT score is <= 0.05, PROVEAN scores is < -2.5. Polyphen predicts a non-synonymous variant as benign, possibly damaging, or probably damaging if score is < 0.2, between 0.2 and 0.85 or > 0.85, respectively. Nucleotide numbering is based on RefSeq DNA accession number NM_152486.2.

Exon	Forward Primer (5'-3')	Reverse Primer (5'-3')	Annealing Temperature (ºC)	Product Size (bp)
SAMD11				
1-2	GACTCAGCCTTTCTGGGC	CTGAAAACCGCAGGGAC	60	483
3	CACCTTCCTCTCCTCCTGC	CCGGTGTGGACACTCTCATC	62	344
4	GCCCTGCTATCCTGAGGC	GAGACAGCGGACAGCAGAC	62	217
5	AGAGCTAGGCACTCCCTGTG	GCTGGTGCTGAGTGGAGAC	62	287
6-7	CAGGCTGAGCTGGAGCAG	GCCTCAGCAACAGGAATGG	60	575
8	CCCCACCTCAGTGTTCTACG	GTGCTGGTGGCTGAGAGC	62	325
9-10	CCGGCAATTAGCGGAGG	CTCTCCAGCTCCTTCTGCC	60	561
11.1	CTGCGGAAGCAGAACCTG	CCCCGTCATCTCCTTGG	60	385
11.2	CTGGTGCTGAACCACGG	AAGACCCTTCCACAGGCG	62	442
12	CACACGACGGTCAGGAGAC	CATCCTTTCCAGGGAGGTAG	62	268
13	CAAACAGCTCCTCTTGGCTC	GTCGCACACCCACCCAG	62	263
14.1[a]	AGAAAGCTCTGGGTGGGTGT	TTCTCCTGCTTGGGTGAAGT	60	243
14.2	TTTTCTACGTGGCCAGCTTC	TGGCTCCTGGAGATTTGTGT	62	269
RNA_12-14	GAGAGTACACTCGGGTCTTCAG	TTCTCCTGCTTGGGTGAAGT	60	316
PLEKHN1				
10 [b]	TTCCTGATTGAAGGTAGGGC	ACGTCGGTCAGGCTGATCTC	60	446

Supplementary Table S6. Primers sequences for Sanger sequencing and expression studies. Oligos were designed using Primer 3 software. [a,b] Oligonucleotides used to verification of the exome variants c.1888C>T; p.R630* and c.995G>A;p.Gly332Glu in *SAMD11* and *PLEKHN1*, respectively.

SNOW test

Clustering coefficient larger than expected by chance (*p-value*= **0.0022**)

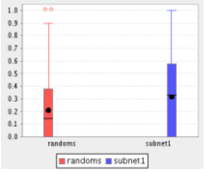

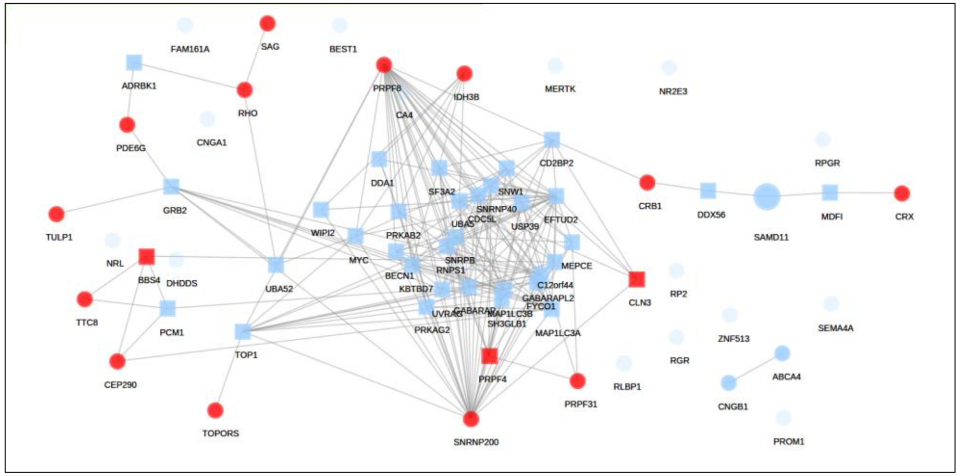

Supplementary Figure S1. Protein-protein network analysis using SNOW tool for *SAMD11* and other retinal disease candidate genes. A significant highly connected network of retinal proteins was obtained including *SAMD11* and several others retinal dystrophy-associated genes. Circles represent the candidate RD genes that are used as input and squares show external nodes that inform about new functional candidates. Each edge indicates the connection between two nodes. Red nodes (circles or squares) represent known genes that have been previously implicated in retinal dystrophy in humans.

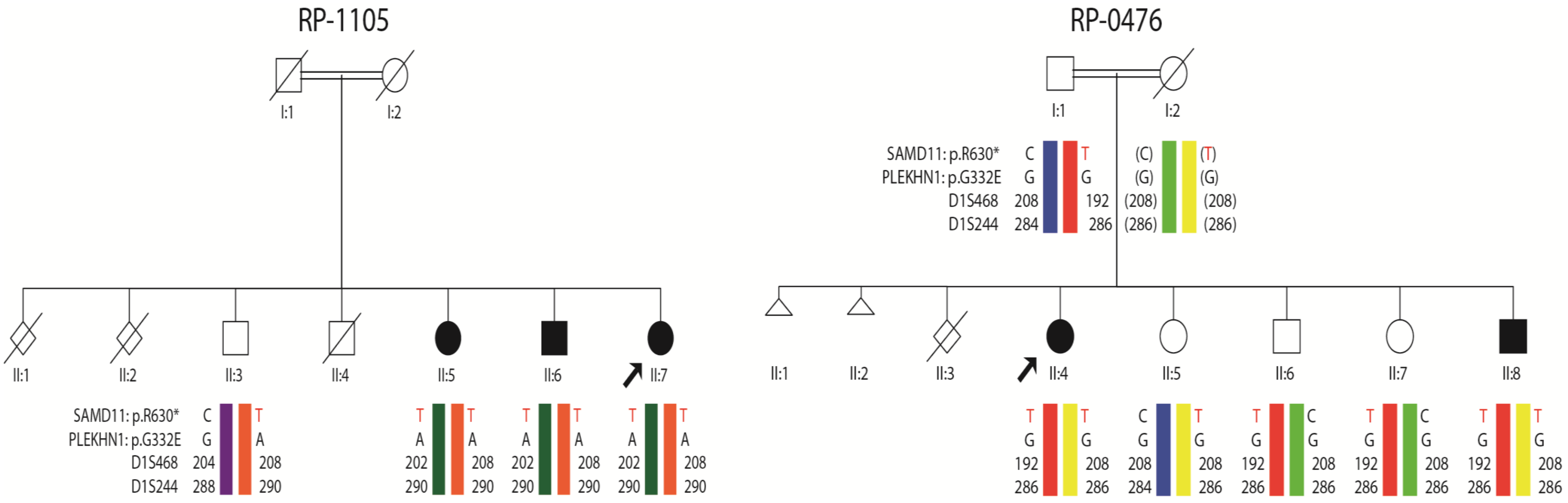

Supplementary Figure S2. Haplotype analysis of in families carrying the mutation c.1888C>T; p.Arg630* in *SAMD11*. Haplotype for telomeric region of the short arm of chromosome 1 was constructed using genotypes from the *SAMD11* mutation (chr1p36.33; chr1:879,375), the rare variant c.995G>A; p.Gly332Glu (chr1:907,797) in *PLEKHN1* found in this study, that is also located at chr1p36.33, only 28kb downstream to *SAMD11*, and two informative polymorphic microsatellites at chr1p36.22, D1S468 (chr1: 3,584,862-3,585,185) and D1S244 (chr1:10,574,064-10,574,379). Mutated allele (T) in *SAMD11* is represented in red. Inferred genotypes for deceased individual I:2 (family RP-0476) were indicated in parentheses.

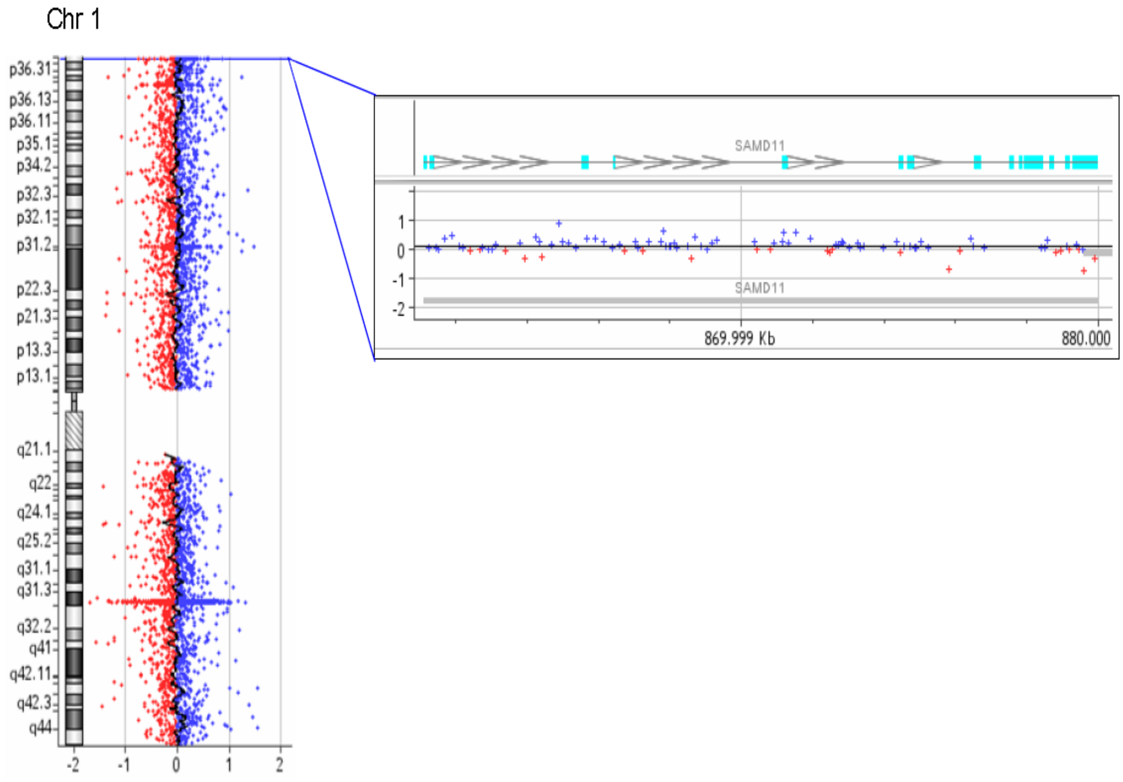

Supplementary Figure S3. Array-based comparative genomic hybridization (aCGH) for *SAMD11*. A custom 8X60k Agilent SurePrint G3 CGH array was designed with an average distribution of 1 probe per 150 bp in the *SAMD11* gene, located at telomere region on short arm of chr1 (1p36.33). A normal pattern of two copies was observed for all 95probes covering the entire genomic region of *SAMD11*. Log-ratio: -2/-1: deletions, 0: normal pattern, +1/+2: duplications.

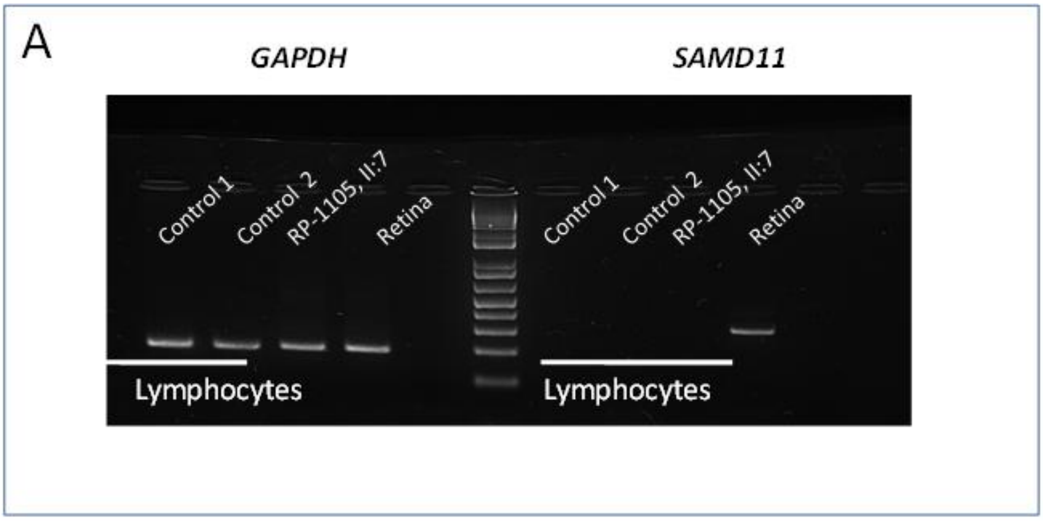

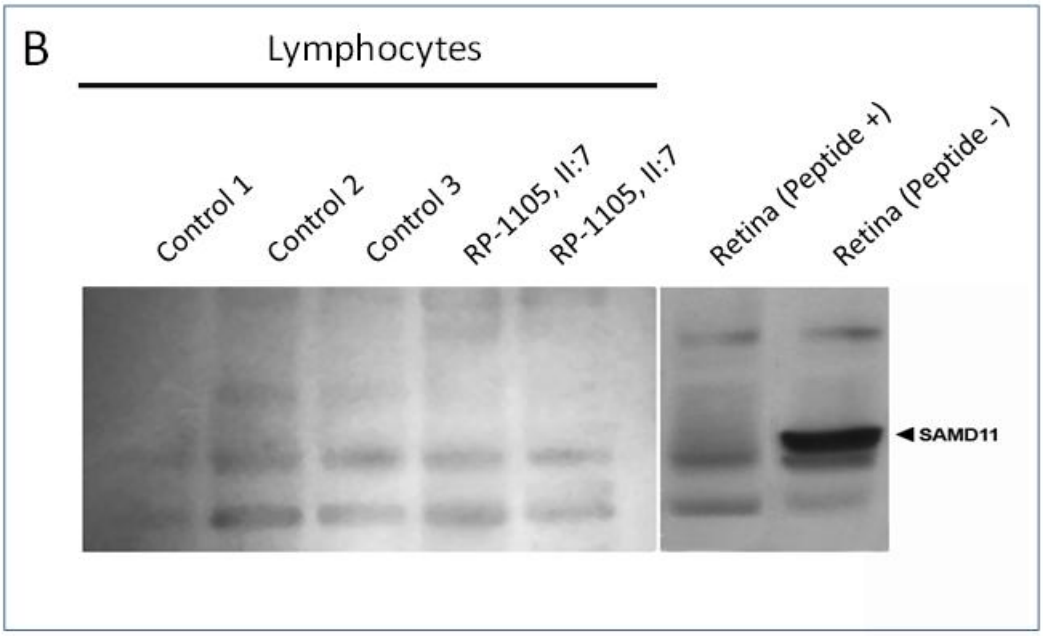

Supplementary Figure S4. SAMD11 expression in EBV-derived lymphoblastoid cell line (LCLs) from controls and the individual II:7 (family RP-1105) carrying the mutation p.Arg330* in *SAMD11*. A. Expression of *SAMD11* by RT-PCR analysis in total RNA from LCLs of two controls and human retina. Amplification of GAPDH mRNA was used as positive control. **B**. Immunoblotting analysis of SAMD11 protein in LCLs. Human retina was used as positive control. The arrowhead point to the 68 kDa protein band corresponding to SAMD11. SAMD11 immunolabeling (peptide -) was specifically abolished when the SAMD11 antibody was preincubated with its immunogen peptide (peptide +).

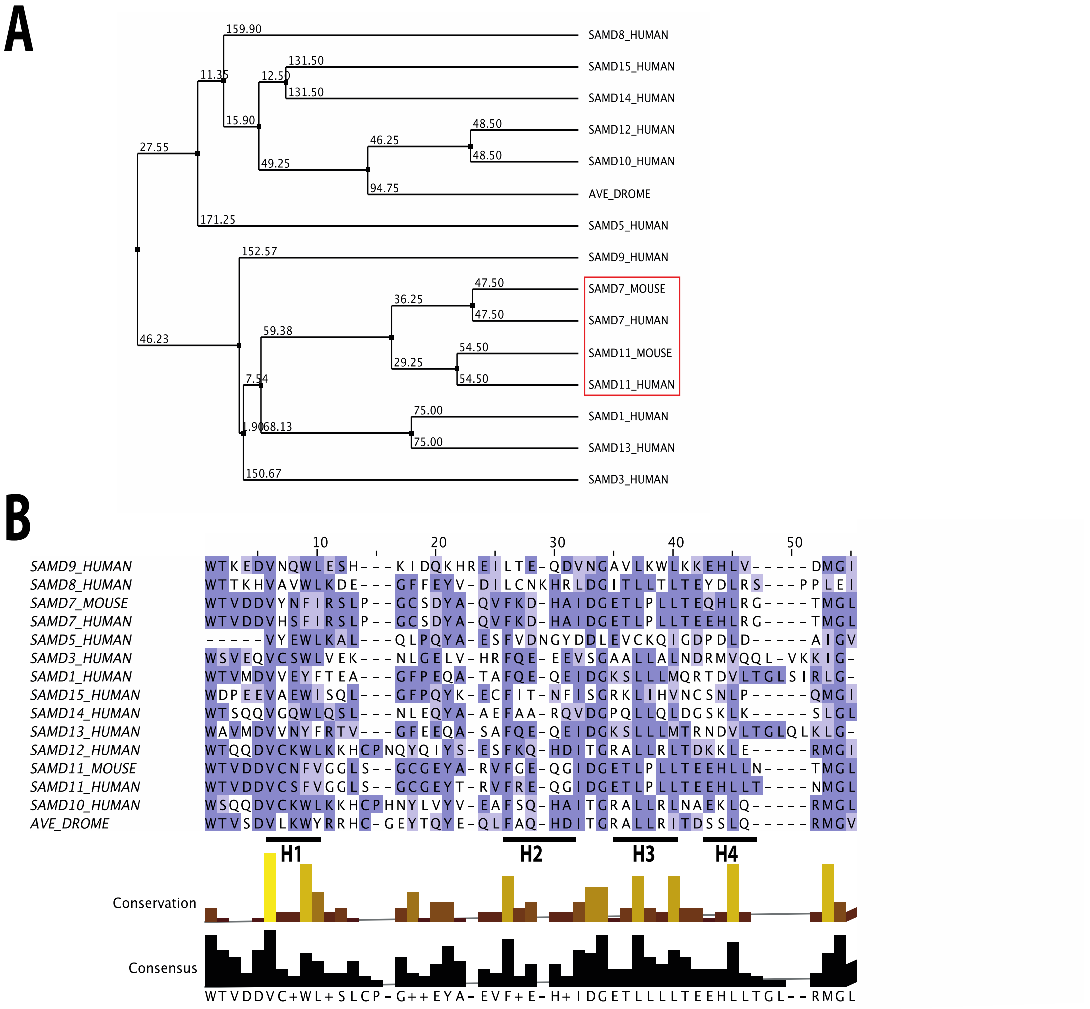

Supplementary Figure S5. Conservation of SAM-domain. (A) Phylogenetic tree of SAM domain containing proteins. Amino-acid sequences were analyzed by the neighbor-joining method using Blosum 62 scores. The branch lengths reflect the mean number of substitution per site that have occurred in that branch and the distance scale represents the number of differences between sequences. **(B).** Amino acid multi-alignment of selected SAM domain sequences for the closest SAM domain-containing proteins using Blosum 62 scores for conservation in the Jalview tool. The level of similarity is indicated by shading ranging from 100% (dark blue) to 60% (light blue). H1-H4 indicates the conserved alpha-helices in SAM domain.

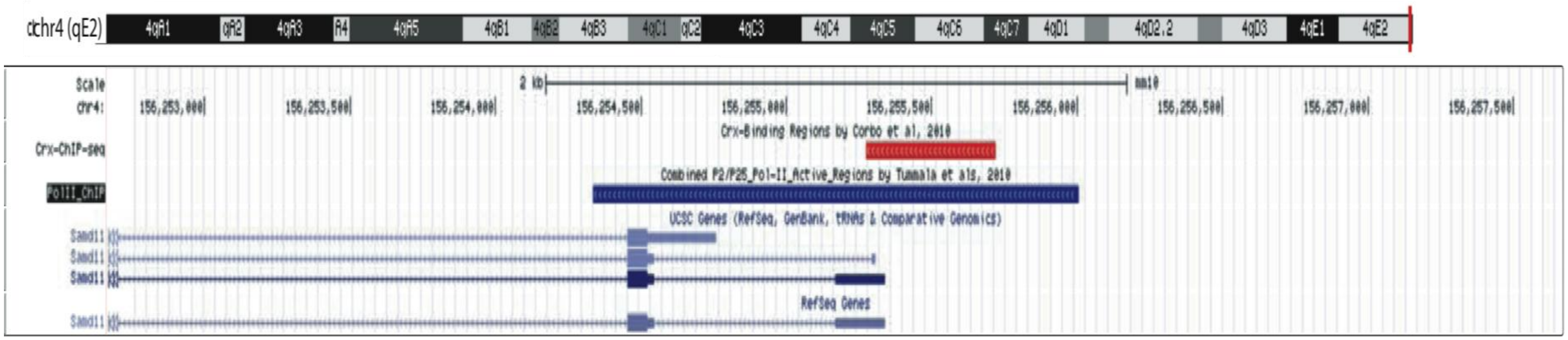

Supplementary Figure S6. EnrichedCrx and RNA polymerase II (Pol II) ChIP-Seq regions are shown in the promoter and first intron of mouse Samd11. Data were inferred from two previously reported ChIP-seq dataset from Corboat, 2010 and Tummala et al, 2010. The original genomic coordinates of ChIP-seq reads from both experiments were lift from mouse assemblies mm9 (July 2007) to mm10 (Dec. 2011) using the UCSC genome browser. Red bar on top represents the genomic localization on mouse assembly mm10 of a putative CBR ranked at position 98 with 154 reads in the original ChIP-seq data (chr4: 155629382-155629830 on mm9). Blue bar represents the genomic localization on mouse assembly mm10 (chr4: 155628446-155630113 on mm9) of an active region for the Pol-II on mouse retina during P2 and P25 developmental stages.
